# Pindel-TD: A Tandem Duplication Detector Based on A Pattern Growth Approach

**DOI:** 10.1093/gpbjnl/qzae008

**Published:** 2024-01-22

**Authors:** Xiaofei Yang, Gaoyang Zheng, Peng Jia, Songbo Wang, Kai Ye

**Affiliations:** School of Computer Science and Technology, Faculty of Electronic and Information Engineering, Xi’an Jiaotong University, Xi’an 710049, China; Center for Mathematical Medical, the First Affiliated Hospital of Xi’an Jiaotong University, Xi’an 710061, China; Genome Institute, the First Affiliated Hospital of Xi’an Jiaotong University, Xi’an 710061, China; MOE Key Lab for Intelligent Networks & Networks Security, Faculty of Electronic and Information Engineering, Xi’an Jiaotong University, Xi’an 710049, China; Center for Mathematical Medical, the First Affiliated Hospital of Xi’an Jiaotong University, Xi’an 710061, China; Genome Institute, the First Affiliated Hospital of Xi’an Jiaotong University, Xi’an 710061, China; MOE Key Lab for Intelligent Networks & Networks Security, Faculty of Electronic and Information Engineering, Xi’an Jiaotong University, Xi’an 710049, China; School of Automation Science and Engineering, Faculty of Electronic and Information Engineering, Xi’an Jiaotong University, Xi’an 710049, China; MOE Key Lab for Intelligent Networks & Networks Security, Faculty of Electronic and Information Engineering, Xi’an Jiaotong University, Xi’an 710049, China; School of Automation Science and Engineering, Faculty of Electronic and Information Engineering, Xi’an Jiaotong University, Xi’an 710049, China; Center for Mathematical Medical, the First Affiliated Hospital of Xi’an Jiaotong University, Xi’an 710061, China; Genome Institute, the First Affiliated Hospital of Xi’an Jiaotong University, Xi’an 710061, China; MOE Key Lab for Intelligent Networks & Networks Security, Faculty of Electronic and Information Engineering, Xi’an Jiaotong University, Xi’an 710049, China; School of Automation Science and Engineering, Faculty of Electronic and Information Engineering, Xi’an Jiaotong University, Xi’an 710049, China; School of Life Science and Technology, Xi’an Jiaotong University, Xi’an 710049, China; Faculty of Science, Leiden University, Leiden 2311 EZ, Netherland

**Keywords:** Tandem duplication, Pattern growth, Short-read sequencing, Structural variation, *SAGE1*

## Abstract

Tandem duplication (TD) is a major type of structural variations (SVs) that plays an important role in novel gene formation and human diseases. However, TDs are often missed or incorrectly classified as insertions by most modern SV detection methods due to the lack of specialized operation on TD-related mutational signals. Herein, we developed a TD detection module for the Pindel tool, referred to as Pindel-TD, based on a TD-specific pattern growth approach. Pindel-TD is capable of detecting TDs with a wide size range at single nucleotide resolution. Using simulated and real read data from HG002, we demonstrated that Pindel-TD outperforms other leading methods in terms of precision, recall, F1-score, and robustness. Furthermore, by applying Pindel-TD to data generated from the K562 cancer cell line, we identified a TD located at the seventh exon of *SAGE1*, providing an explanation for its high expression. Pindel-TD is available for non-commercial use at https://github.com/xjtu-omics/pindel.

## Introduction

Tandem duplication (TD) is one of the major types of structural variation (SV) [1,2], contributing to the formation of *de novo* gene structure [3], the evolution of biosynthetic pathways in plants [4], and various human diseases, including autism [5] and cancers. Cancers such as breast, ovarian, and endometrial carcinomas can be further divided into different tandem duplicator phenotype (TDP) subgroups based on the frequency and distribution of TDs [6,7]. TDs contribute to tumorigenesis by augmenting oncogene expression and disrupting tumor suppressor genes [6,7].

Fueled by the development of next-generation and third-generation sequencing technologies, numerous SV detection methods have been developed based on model-matched or model-free strategies. In general, model-matched strategies involve extracting the mutational signal, such as discordant read-pairs, read-depth, and split-reads, and then matching the signal to a specific SV model. For example, Pindel utilizes a pattern growth approach to leverage split-read signals for precise breakpoint SV detection [8,9], while DELLY performs an integration analysis of the pair-end mapping and split-read information to detect SVs at single nucleotide resolution [10]. LUMPY was developed to precisely identify SVs by integrating three different mutational signals of read-pair, split-read, and read-depth using general probabilistic framework [11]. While model-matched SV detection strategies are efficient in detecting simple SVs, *e.g*., insertion, deletion, and inversion, they often lack performance for complex SVs (CSVs) that contain multiple breakpoints and play important roles in cancer [9,12]. Mako [13] and SVision [14] are two model-free methods designed to detect CSVs from the short-read and long-read sequencing data, respectively. Although these methods perform well for the most frequent types of SVs, such as insertions and deletions, they exhibit lower accuracy and recall rates for certain types of SVs, such as TDs, which are frequently reported as insertions [15,16] due to the lack of specific optimization on TDs. Therefore, developing a TD detection tool specifically optimized to accurately characterize TDs is an eminent need in the genomic community.

Here, we reported Pindel-TD, a TD detection module of the Pindel method, in which we specifically optimized the pattern growth approach applied in Pindel to accommodate the short-read alignment signatures of TDs across a wide size range. We also applied split-read analysis in Pindel-TD to acquire single nucleotide resolution of the breakpoint. We evaluated the performance of Pindel-TD on both simulated sequencing data and real sequencing data of the sample HG002 [17], demonstrating that Pindel-TD is effective for TD detection and outperforms other methods, *e.g.*, Manta [18], DELLY [10], LUMPY [11], and DINTD [19]. We also report the results of Pindel-TD using short-read sequencing data of the K562 cancer cell line from Encyclopedia of DNA Elements (ENCODE) [20] to illustrate its potential application in cancer genomic data.

## Method

### Pindel-TD

#### Overview

**Figure 1:**
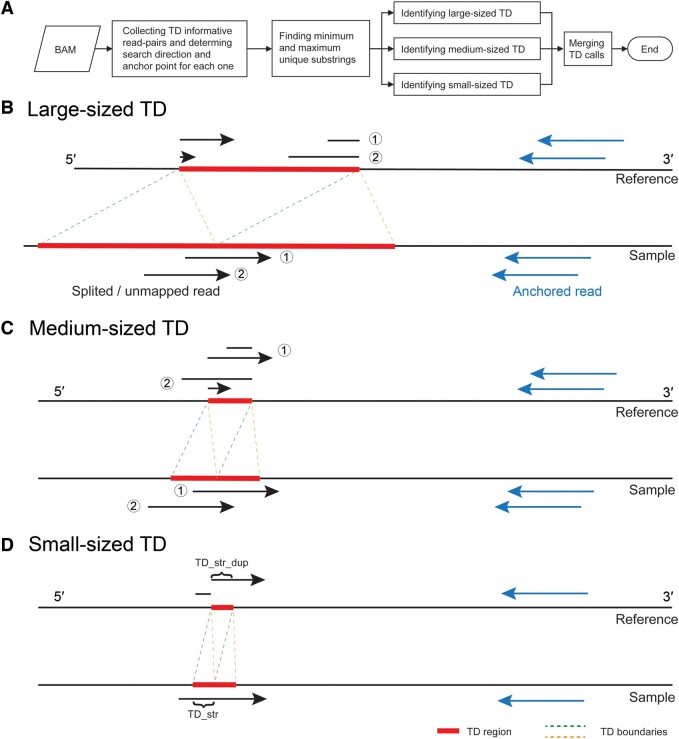
Pindel-TD framework **A**. The workflow of Pindel-TD. Characterization of the alignment signals for large-sized TDs (**B**), medium-sized TDs (**C**), and small-sized TDs (**D**). For each TD informative read-pair on reference, two segments of the unmapped reads indicate the detected minimum and maximum substrings used to determine the breakpoint. “TD_str” and “TD_str_dup” indicate the TD sequences inferred from unmapped reads. TD, tandem duplication.

We proposed a pattern growth approach for TD detection with different sizes and implemented Pindel-TD in C++. Overall, there are four steps for TD detection (**Figure 1**A), following the framework of pattern growth strategy in Pindel for the detection of large deletion and small insertion [8]. Firstly, we selected read-pairs with only one uniquely mapped read [mapped only with the “M” character in its Concise Idiosyncratic Gapped Alignment Report (CIGAR) string] and its mate showing split-read (soft-clipped) characteristics. For each selected read-pair, the mapped read with high mapping quality (larger than the “anchor_quality” parameter, default is 30) was considered as the reliable anchor read (*e.g.*, blue arrows in Figure 1B–D), guiding subsequent split-read analysis of soft-clipped reads. We then applied a pattern growth approach to determine the minimum (the “min_distance_to_the_end” parameter in Pindel-TD, default is 8) and maximum unique substrings starting from either the leftmost or the rightmost of the unmapped read [showing the soft-clipped reads in Binary Alignment Map (BAM) file, *e.g.*, black arrows in Figure 1], followed by careful processing of split-read information to identify the TDs with accurate breakpoints. Finally, the redundant TDs were removed according to their length and breakpoints to obtain the final TD set.

#### Detailed steps

We defined the size of TDs as follows: len(TD)>read_len for large-size, read_len>len(TD)>1/2×read_len for medium-size, and len(TD)<1/2×read_len for small-size, respectively, where *read_len* indicated the read length, and *len*(*TD*) indicated the length of a TD event.

We designed four steps for detecting TDs across a wide size spectrum (Figure 1). (1) For each TD informative read-pair, the anchor point was defined as the 3′ end of the mapped read. (2) The pattern growth approach was employed to search for minimum and maximum unique substrings from the 3′ end of unmapped read within a specific range (two times the insert size from the anchor point). (3) Similarly, the pattern growth approach was utilized to search for both minimum and maximum unique substrings from the 5′ end of unmapped read within a specified region. The parameters defining the region were contingent upon the size of TD. For large-sized TDs, the region was defined as a range of “Max_TD_Size” (a user-defined parameter) from the previously mapped 5′ end of the unmapped read obtained in step 2. For medium-sized and small-sized TDs, the region was delineated as the range of two times “Max_Span_Size” (a user-defined parameter) with the center being the previously mapped 5′ end obtained from step 2. In the case of small-sized TDs, the search was terminated once the current search coordinate matched the coordinate of the previously mapped 5′ end obtained from step 2. (4) For large-sized and medium-sized TDs, the substrings extracted from step 2 and step 3 were combined to reconstruct a complete unmapped read. If succeeded, Pindel-TD recorded two substring ends as breakpoints of a large-sized TD and stored the interval in the candidate TD database. For small-sized TDs, Pindel-TD extracted the candidate duplication sequence by cutting an equal length of sequence from the maximum unique substring obtained in step 2 at 5′ end (“TD_str_dup” in Figure 1D) and calculated the edit distance between “TD_str” and “TD_str_dup” by Edlib (https://github.com/Martinsos/edlib) [21] to determine reliability. If the edit distance was smaller than 4, Pindel-TD stored it in the database.

All records in the candidate TD database were sorted by the 5′ reference coordinate, and those with at least two supported reads were considered as reliable TDs.

#### Merging TD calls

Given the potential overlap in alignment signals from the aforementioned three strategies, it is necessary to merge TD calls. For a TD call $TD_{i}$, we identified its overlapping TD callset by checking whether the range of a TD call ($TD_{j}$) overlapped with $TD_{i}$ and $\left|\mathrm{len}\left(TD_{i}\right)-\mathrm{len}\left(TD_{j}\right)\right|<2\times \min\left(\mathrm{len}\left(TD_{i}\right),\mathrm{len}\left(TD_{j}\right)\right)$, where $\mathrm{len}\left(TD_{i}\right)$ was the length of $TD_{i}$. Within the identified set, we selected the most frequent TD call as the representative TD event and reported it. This process was executed iteratively to obtain the final TDs.

### Simulated data generation

To evaluate the performance of Pindel-TD, we simulated different sizes of TDs on human chromosome 1 by VISOR (v1.1) [22]. We first applied the “randomregion.r” script in VISOR to generate 37 Browser Extensible Data (BED) files on human chromosome 1 (Table S1). Each BED file contained 100 simulated TDs with a specific length varying from 10 bp to 9 kb (Table S1). Then the “VISOR HACk -b $TD_BED -g human_chr1.fa -o $OUTPUT” command was used to incorporate the simulated TDs into human chromosome 1 of GRCh38. Following this, we employed wgsim (v1.12; https://github.com/lh3/wgsim) to simulate 15× coverage of paired-end sequencing data for the human chromosome 1 containing simulated TDs with 100-bp read length and 400-bp insert size, with an error rate set as 0.5%. The simulated sequencing data were aligned to GRCh38 chromosome 1 by BWA-MEM (v0.7.17-r1188) [23] to gain the BAM file for TD calling. Furthermore, we applied BEDTools intersect to verify the recovery of simulated TDs by different methods.

### Processing the benchmark SV set of real data (HG002/NA24385)

The benchmark SV set (v0.6) of HG002/NA24385 [17] was constructed by the integration of SV callsets from 19 SV detection methods and 4 sequencing technologies, including short-read, linked-read, long-read, and Bionano optical map, against GRCh37 [17]. In this benchmark, the SVTYPE only labeled INS and DEL for insertions and deletions, respectively, while the duplications (both tandem and dispersed duplications) were reported as DUP in REPTYPE. A total of 2817 duplications were identified, 1882 of which were labeled as Illcalls (calling from Illumina short-read sequencing data).

To identify TDs from the duplications, we manually inspected the dot plots created by Gepard (v2.1.0) [24] using the DUP-related long-reads extracted from the BAM file generated by NGMLR [25] with parameter “-x pacbio” based on the high-fidelity reads and the related GRCh37 sequences. Furthermore, candidate TDs located at repetitive regions of GRCh37 were excluded, since detection of TDs in these regions remains challenging for short-read-based methods [1]. Eventually, we obtained 600 non-repetitive benchmarked TDs (Table S2). We applied the Truvari bench subcommand with parameters “-r 1000 -p 0.0 -P 0.5 -s 50 -S 50 --sizemax 2000 --typeignore” (v3.5.0) [26] to compare the TD calls reported by various tools with the benchmark.

## Results

### Performance evaluation on simulated data

**Figure 2:**
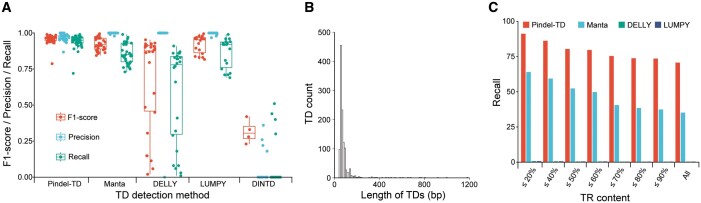
The performance evaluation on simulated and real data **A**. The F1-score, precision, and recall on the simulated data for four TD detection methods. The detailed values are shown in Table S3. **B**. The length distribution of 1162 TDs detected from HG002 sequencing data. **C**. The recall of four methods on HG002 benchmarked TDs for different TR contents. TR, tandem repeat.

To assess the performance of Pindel-TD on TD detection, we conducted a comprehensive evaluation by comparing its recovery of simulated TDs with that of Manta, DELLY, LUMPY, and DINTD. Precision, recall, and F1-score were calculated for each TD set with different sizes (Table S3). We found that Pindel-TD achieved the highest performance on simulated TDs, achieving average precision, recall, and F1-score of 97%, 94%, and 96%, respectively. In comparison, Manta, LUMPY, DELLY, and DINTD followed with average F1-scores of 92%, 92%, 68%, and 31%, respectively (**Figure 2**A; Table S3). The average false discovery rate of Pindel-TD was approximately 3% (Table S3). The most significant improvement of Pindel-TD over other methods was the detection of TDs with lengths smaller than the read length of 100 bp (Table S3). In addition, Pindel-TD extended the lower bound of the detectable TDs from 30 bp (Manta) to 10 bp, achieving an average F1-score of 92% for TDs smaller than 30 bp (Table S3). In contrast, DELLY and LUMPY demonstrated limitations in detecting small-sized TDs, with DELLY missing all TDs smaller than 60 bp and LUMPY missing all TDs smaller than 100 bp. For TDs larger than 500 bp, Manta obtained a slightly higher average F1-score than Pindel-TD (97% for Manta and 95% for Pindel-TD), possibly due to the decreased probability of finding a unique substring when enlarging the parameter “Max_TD_Size” in step 3. Overall, the simulated evaluation results indicate that Pindel-TD is an effective and robust method for detecting TDs across the entire size range.

### Performance evaluation on HG002 data

We further examined the performance of Pindel-TD on paired-end sequencing data from the well-characterized cell line HG002/GM24385, as provided by the Genome in a Bottle project [17]. We applied Pindel-TD on the short-read sequencing data of HG002 and detected 1162 TDs with a length greater than 50 bp (Figure 2B; Table S4). We found that 955 of the 1162 TDs (82.2%) overlapped with the released benchmark SV set (v0.6), higher than that from Manta (632 of 799, 79.1%), DELLY (390 of 1032, 37.8%), and LUMPY (109 of 227, 48.2%) (Table S5). Moreover, Pindel-TD demonstrated the recall of 70.5% of benchmarked TDs, outperforming Manta (35.0%), DELLY (0.2%), and LUMPY (0.2%) (Figure 2C; Table S6), indicating its effectiveness in TD detection. It is worth noting that the presence of the reference tandem repeat (TR) sequence can introduce TD-like alignment signals, potentially affecting the accuracy of TD prediction (Table S6). We subsequently investigated the influence of TR content on the detection of TDs for these four methods. We computed the TR content of each benchmarked TD by Tandem Repeat Finder (TRF) [27] and calculated the recall for different TR contents. Our analysis revealed that Pindel-TD exhibited a recall ranging from 91.0% to 73.4% as TR content varying from ≤ 20% to ≤ 90%, while the second-best performing method, Manta, showed a recall ranging from 63.8% to 37.2% under similar TR content variations (Figure 2C; Table S6), indicating the robustness of Pindel-TD over other methods in different TR content scenarios.

### Detecting TDs in K562 cell line

**Figure 3:**
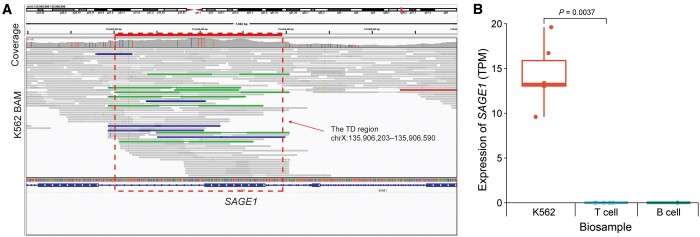
A *SAGE1*-related TD detected from K562 cell line **A**. The IGV screenshot of a TD located at chrX:135,906,203–135,906,590 of the K562 cell line. The red dashed box indicates the location of TD. **B**. The gene expression of *SAGE1* in K562 cells, T cells, and B cells. The *P* value was calculated by Wilcoxon rank-sum test and Wilcoxon signed-rank test. The *P* value between the expression levels of K562 and B cells was not able to be calculated due to an insufficient number of data points (only one RNA-seq data of B cells acquired from ENCODE). IGV, Integrative Genomics Viewer; RNA-seq, RNA sequencing; TPM, transcript per million.

To test the potential usage of Pindel-TD in analyzing cancer genomic sequencing data, we applied it to the short-read sequencing data of the human erythroleukemic cell line K562 from ENCODE [20]. In total, we detected 888 TDs on autosomes and chromosome X, and 531 out of them were Pindel-TD-specific TDs (Table S7). Part of the detected TDs were related to protein-coding genes, *e.g.*, *FLT3*, *MUC3A*, *SAGE1*, *PLIN4*, *CBS*, and *ACSF3*. Specifically, we identified one TD (chrX:135,906,203–135,906,590) occurring on the seventh exon of *SAGE1*, a gene encoding a cancer antigen (**Figure 3**A). The expression of *SAGE1* in K562 cell line was significantly higher than that in T cell (*P* value = 0.0037, Wilcoxon rank-sum test and Wilcoxon signed-rank test) (Figure 3B; Table S8), with the seventh exon acquiring the highest read coverage among its 20 exons (Figure S1). These findings suggest that TD directly contributes to increased gene expression of *SAGE1* in K562 cell line. Notably, the tumor-specific expression pattern of *SAGE1* has been found in several solid cancers, *e.g.*, bladder cancer, lung cancer, and head and neck cancer, highlighting its role as a potential target for cancer immunotherapy [28]. A few studies have also reported the relevance between *SAGE1* and leukemia [29,30], while the genetic variation of *SAGE1* has not been well studied in cancers. One of the Pindel-TD-specific TD (chr21:43,063,062–43,063,130) affects the coding sequence of the protein-coding gene *CBS*, which encodes cystathionine β-synthase. Higher expression levels of *CBS* in chronic myeloid leukemia (CML) patients than that in control samples have been reported [31], yet the underlying genetic mechanism remains poorly understood. Our findings provide a possible explanation for its higher expression in CML. Taken together, these results underscore the potential of TD module in Pindel for the analysis of cancer genomic sequencing data.

## Discussion

In this study, we developed Pindel-TD, a TD detection model, by specifically optimizing the pattern growth approach in Pindel. We refined the search strategies for minimum and maximum unique substrings to accommodate TDs of different sizes, resulting in high and robust performance in detecting TDs across a wide size range. Comparison with widely-used SV detection methods, including Manta, DELLY, and LUMPY, on simulated and HG002 sequencing data demonstrated the outperformance of Pindel-TD. We further applied Pindel-TD on the sequencing data of the K562 cell line and detected a TD occurring on the seventh exon of *SAGE1*, which potentially explained the high expression of *SAGE1*. This highlights the potential application of Pindel-TD in cancer studies.

We evaluated the false discovery rate based on the simulated data (Table S1). However, it is challenging to evaluate the false positive rate due to the lack of negative TDs for either simulated or real data. Albeit split-reads were used to improve the breakpoint resolution, they only accounted for a small part of the total reads related to TDs, limiting genotype analysis.

Currently, Pindel is developed for short-read sequencing data and does not support the data from long-read sequencing, despite being selected as the Method of Year 2022 [32]. While long-read sequencing has empowered genomic studies, *e.g.*, genome assembly [33–36] and SV detection [37,38], its high cost limits the accessibility to broader research communities, *e.g*., large cohort and clinic genomics studies. Therefore, methods developed for short-read sequencing data continue to hold irreplaceable value for genomic community. In addition, we are trying to develop a new version of Pindel to support both long-read and short-read sequencing data for detecting both simple and complex SVs.

## Code availability

Pindel-TD is available at https://ngdc.cncb.ac.cn/biocode/tools/BT007384 and https://github.com/xjtu-omics/pindel. It is free for non-commercial use by academic, government, and non-profit/not-for-profit institutions. A commercial version of the software is available and licensed through Xi’an Jiaotong University. For more information, please contact kaiye@xjtu.edu.cn.

## CRediT author statement

**Xiaofei Yang:** Methodology, Software, Validation, Visualization, Investigation, Resources, Writing – original draft, Writing – review & editing. **Gaoyang Zheng:** Methodology, Software, Data curation, Validation, Writing – original draft. **Peng Jia:** Methodology. **Songbo Wang:** Methodology. **Kai Ye:** Conceptualization, Supervision, Project administration, Writing – review & editing. All authors have read and approved the final manuscript.

## Supplementary material

Supplementary material is available at *Genomics, Proteomics & Bioinformatics* online (https://doi.org/10.1093/gpbjnl/qzae008).

## Competing interests

The authors have declared no competing interests.

## Supplementary Material

qzae008_Supplementary_Data
