# Supplementary material for: Pindel-TD: A Tandem Duplication Detector Based on A Pattern Growth Approach
Source: Genomics Proteomics Bioinformatics. 2024 Jan 22;22(1):qzae008. doi: 10.1093/gpbjnl/qzae008 (PMC11425056; doi:10.1093/gpbjnl/qzae008)
Supplement: qzae008_Supplementary_Data [file qzae008_supplementary_data.zip › Supplementary captions.docx]

# Supplementary material

**Figure S1 The comparison of RNA-seq coverage of *SAGE1* in K562 cell line, T cells, and B cells**

The RNA-seq coverage of *SAGE1* in K562 cell line under ENCODE accession of ENCSR062FHL (**A**), ENCSR601DZY (**B**), and ENCSR100JNS (**C**). **D.** The RNA-seq coverage of *SAGE1* in T cells under ENCODE accession of ENCSR100JNS. **E.** The RNA-seq coverage of *SAGE1* in B cells under ENCODE accession of ENCSR896YYL. The red dashed boxes indicate the TD located at chrX:135,906,203–135,906,590.

**Table S1 The simulated TDs on GRCh38 chromosome 1**

**Table S2 The benchmarked non-repetitive TDs in HG002**

**Table S3 The performance evaluation of SV methods on simulated TDs**

**Table S4** **The 1162 TDs detected by Pindel-TD from HG002**

**Table S5 The precision of detecting TDs on HG002 sequencing data**

**Table S6 The recall of different methods on HG002 benchmarked TDs for different TR contents**

**Table S7 The TDs detected from K562 cell line**

**Table S8 The gene expression of *SAGE1 in* K562 cell line, T cells, and B cells from ENCODE**
