# Supplementary figures and images for: Pindel-TD: A Tandem Duplication Detector Based on A Pattern Growth Approach
Source: Genomics Proteomics Bioinformatics. 2024 Jan 22;22(1):qzae008. doi: 10.1093/gpbjnl/qzae008 (PMC11425056; doi:10.1093/gpbjnl/qzae008)

## Slide 1
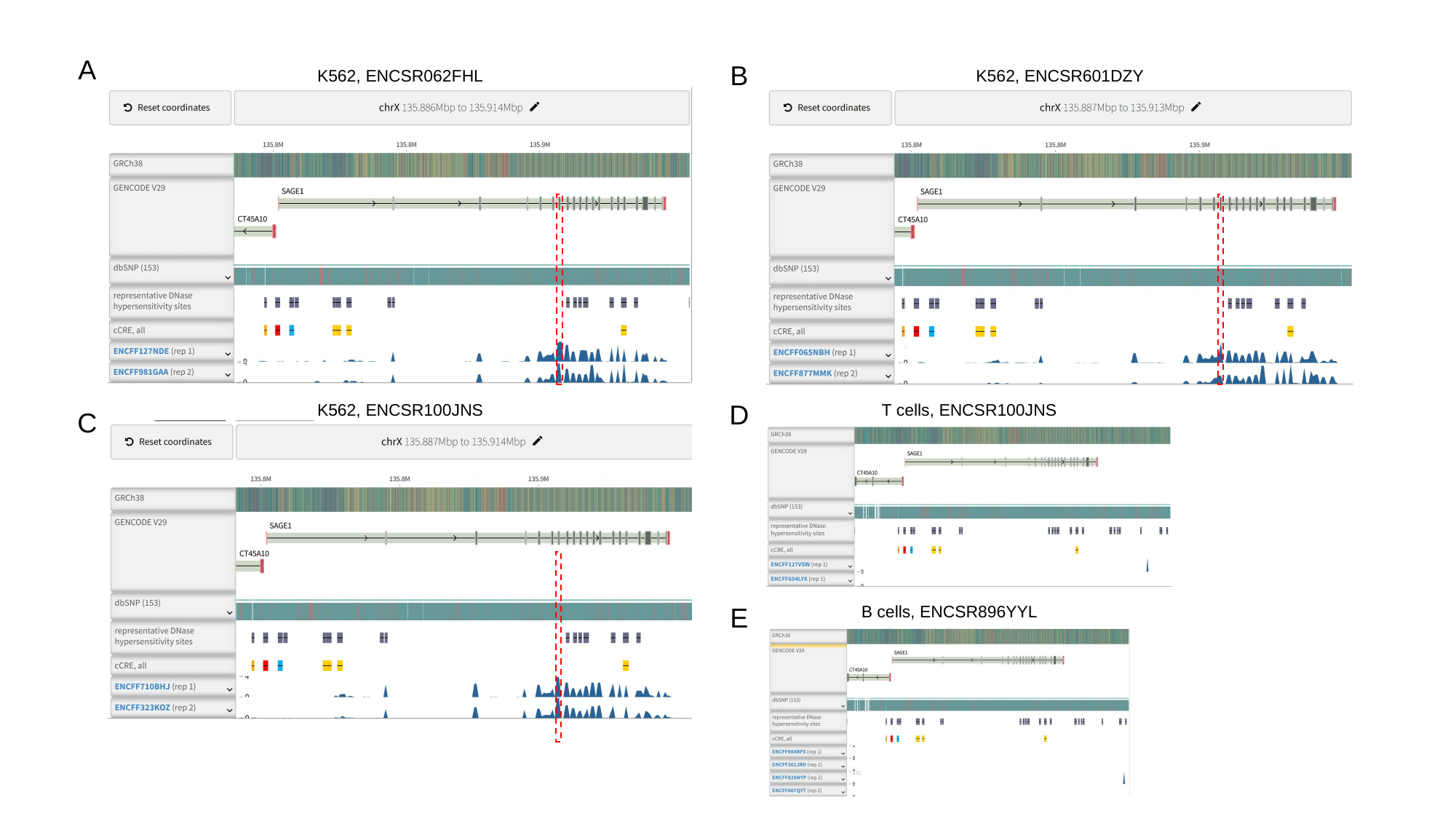

A
B
K562, ENCSR062FHL
K562, ENCSR601DZY
D
T cells, ENCSR100JNS
K562, ENCSR100JNS
C
E
B cells, ENCSR896YYL

Supplement: qzae008_Supplementary_Data [file qzae008_supplementary_data.zip › Figure S1-confirm.pptx]
